# Enhancing global health diplomacy for non-communicable diseases: application of the global health network framework

**DOI:** 10.1186/s12992-023-00944-7

**Published:** 2023-06-21

**Authors:** Mahnaz Afshari, Ahmad Ahmadi Teymourlouy, Mohammadreza Maleki, Mohsen Asadi-Lari

**Affiliations:** 1 Social Determinants of Health Research Center, Saveh University of Medical Sciences, Saveh, Iran; 2 Department of Health Service Management, School of Health Management and Information Sciences, Iran University of Medical Sciences, Tehran, Iran; 3 Department of Epidemiology, School of Public Health, Iran University of Medical Sciences, Tehran, Iran

**Keywords:** Global health diplomacy, NCDs, Global health network

## Abstract

**Background and aim:**

Understanding the characteristics of global policy actors and the political context in which they address diplomatic issues in the field of NCDs can play an important role in advancing NCD-related goals. The purpose of this study was to identify and analyze the network of global health actors in the field of NCDs in Iran.

**Methods:**

This study was conducted in 2020 using a qualitative methodology and framework analysis. In-depth semi-structured interviews were conducted with subject-matter experts from all levels of diplomacy, including global, regional, and national, who had managerial, administrative, and academic experience.

**Findings:**

A total of 21 interviews were conducted with individuals who met the inclusion criteria. Following the framework of the World Health Network, the findings were divided into three general areas: the features of the network and the actors; the policy environment; and the characteristics of the issue.

**Conclusion:**

A successful and sustainable program to combat NCDs requires the participation of multiple actors from governments, the private sector, and civil society at international, national, and local levels. The Global Network for Prevention and Control of NCDs should enhance the effectiveness of NCDs policies by highlighting the need to simultaneously improve the internal factors of the network, including relationships among the actors; external factors, including the policy environment; and the complex nature of NCDs.

**Supplementary Information:**

The online version contains supplementary material available at 10.1186/s12992-023-00944-7.

## Introduction

NCDs cause 41 million deaths worldwide each year, equivalent to about 71% of all deaths globally, about 85% of which occur in low- and middle-income countries [1]. This highlights the need to pay closer attention to NCDs in developing countries. Due to the increasing mortality rates from NCDs, especially in developing countries, the World Health Organization (WHO) has declared NCDs a health priority in these countries for the last two decades.

Iran is one of the major countries in the region where improved health conditions, changing socioeconomic status, and unplanned urbanization has led to a shift in the burden of disease from communicable diseases to NCDs [2]. The rising deaths and disability-adjusted life-years (DALYs) over the last few decades are a formidable threat to Iran. NCDs not only lead to early death but also cause significant disability [3]. The occurrence of 6.5 million years of lost life (YLLs) and 8.2 million years lived with disability (YLDs) indicate the growing burden of NCDs in Iran [4]. Sustainable Development Goal Target 3.4 calls for reducing premature death from NCDs. As such, Iran, like other countries that have participated in WHO Global NCDs Action Plan, is tasked with reducing premature deaths from NCDs by 25% by 2025 [5].

Due to the major problems in NCDs prevention and control, the Iranian Ministry of Health launched a nationwide NCDs care system in 2004 [6] and according to the National Policy for Prevention and Control of NCDs, with strategic goals consisting of behavioral factors (e.g., reducing tobacco use, salt intake, physical inactivity) and biological risk factors (e.g., reducing the prevalence of obesity, diabetes, high blood pressure, depression, dental caries) [7]. In line with the implementation of these policies, many activities have been carried out to prevent and control NCDs including establishing National Non-Communicable Diseases Committee (INCDC) through multi-sectoral mechanisms and compiling the national document for the prevention and control of NCDs and related risk factors in the Islamic Republic of Iran from 2015 to 2025 [8], calculating National and Sub-national Burden of Disease (NASBOD) [9], using the WHO STEPwise approach to Surveillance of NCD risk factors (STEPS)[10], adapting “package of essential NCDs’ interventions for primary health care (PHC) in low-resource settings” and conducting the IraPEN study [11, 12], assess the Return on Investment (ROI) for implementation of NCDs prevention program in Iran [13].

As a results of INCDCs performance, the WHO recognized Iran as a fast-track country in 2016, and also the United Nations Interagency Task Force on NCDs (UNITAF) awarded it in 2018 [14]. The WHO’s NCDs progress monitor show that Iran achieved 14 of 19 indicator completely, achieved 3 of 19 partially and not achieved to 2 indicator in 2022 [15]. Increased excise taxes and prices in tobacoo demand-reduction measures, public education and awareness campaign on physical activity, mass media campaigns, marketing of breast-milk substitutes restrictions are the main challenges, which in addition to technical aspects, requires political negotiations and solutions. Global Health Diplomacy (GHD), which involves the participation of a wide range of actors and stakeholders, can be crucial in this regard [15, 16]. An effective response to NCDs requires a multisectoral approach at different levels within and outside the health sector. By building stronger diplomatic relations and collaboration among non-governmental organizations (NGOs), private firms, and governments, various mechanisms can be employed, including increasing public funding, ensuring the supply of essential medicines, investing in hospitals or equipment, and training health professionals and As a result, the way to advance the goals of the non-communicable diseases program in Iran is paved [5, 6, 17].

GHD, if done right, will improve global health and lead to greater equity, better relations between states, and a stronger stakeholder commitment to work together to improve health nationally and globally. Policy interventions for the prevention and control of NCDs must begin with diplomatic negotiations between state officials. In particular, the use of health diplomacy in multilateral negotiations can help tackle health crises. The growing trend of health threats such as the spread of chronic and NCDs worldwide, Increasing prevalence of unhealthy lifestyles and unhealthy products and goods in the whole world and increasing the power of multinational corporations of harmful goods including tobacco highlight the need for diplomats who do not only understand health issues but also can negotiate effectively in a multinational foreign policy environment [16, 18, 19].

Over the recent decades, global health networks have grown rapidly and now exist for most conditions that cause a high disease burden such as NCDs in low-income countries. however, there is not enough information about how networks emerge and their effects on the world [20]. A proper understanding of the characteristics of key actors and the global political context in which they address diplomatic issues in the field of NCDs can play an important role in advancing the goals set for NCDs prevention and control. Therefore, the purpose of this study is to identify and analyze the network of global health actors, in the field of NCDs in Iran.

## Methods

This study was conducted in 2020 using a qualitative methodology and framework analysis, which is a type of qualitative content analysis that summarizes data thematically to facilitate data analysis. The stages in framework analysis are as follows [21]: (1) transcription (done immediately after interviews); (2) familiarization with the interview (reading the transcripts several times and being immersed in the data); (3) coding (identifying and extracting the initial codes); (4) developing a working analytical framework (generating codes several times and comparing the codes of each researcher); (5) applying the analytical framework (indexing); (6) charting data into the framework matrix; and (7) interpreting the data.

The data were collected through in-depth, semi-structured interviews. A purposeful, maximum variation sampling was used for the participants’ recruitment from a broad range of experience and knowledge [10]. Individuals were included from different levels of policymaking and policy implementation within the fields of health diplomacy, global health, and NCDs, as well as organizations and interest groups with at least three years of relevant work experience. It was followed by a snowballing method in which participants recommended others who were eligible and informed to participate in this study.

In total, 21 interviews were conducted with the Deputy Minister of Health in charge of the NCDs Department; the International Relations Department of the Ministry of Foreign Affairs; the Director of the NCDs Department at the Ministry of Health; Iranian health representative in Geneva; WHO representative in Iran; Iranian ambassadors to European Countries; members of the team in charge of developing the National NCDs Prevention and Control Document; WHO staff (WHO office at the Ministry of Health, WHO regional office, and WHO headquarters in Geneva); and officials, experts, and researchers in the fields of NCDs, global health, and GHD. The purposive selection of key informants allows us to access rich data and gain a better understanding of the subject matter.

An interview guide was developed based on a review of the literature and according to the objectives of the research. This guide was used in three pilot interviews with experts and any flaws were eliminated using the feedback from the panel. Interviews were conducted by appointment at the participant’s workplace or via zoom/WhatsApp video calls. The interviewees’ quotations were marked with the letter “I” in this article. If needed, the transcript of the interviews was sent to the interviewees for verification and to add or subtract while confirming. All interviews were typed verbatim. The transcripts were 9 to 18 pages long.

Data analysis was performed concurrently with data collection. Immediately after each interview and before starting the next one, the recorded audio was transcribed, and MAXQDA 10 was used for handling and coding the data. Each word and phrase in the transcript was considered a unit of analysis. Taking notes while re-reading the transcripts helped identify the initial connection between the concepts extracted from the statements. The notes and codes helped the themes to emerge, and as the interviews progressed and the connections between the themes became more clear, it became possible to identify the main patterns and meanings within the interviews. To evaluate the accuracy of the coding process, the research team reviewed the transcripts, and to check their precision, the transcripts were also presented to the participants. The research method included recording all the interviews, transcribing them verbatim, storing them on a computer, becoming immersed in the data, and coding.

Each interview was entirely audio-recorded with the consent of the participants. Then, it was transcribed verbatim without any changes, and finally, the data were analyzed. Various measures were taken to ensure the validity of the data (credibility, transferability, dependability, and confirmability), such as obtaining additional comments from two colleagues and a specialist familiar with qualitative research, sending the transcripts and extracted codes to each participant for verification, performing analysis concurrently with data collection, and transcribing the interviews at the earliest possible opportunity.

**Fig. 1:**
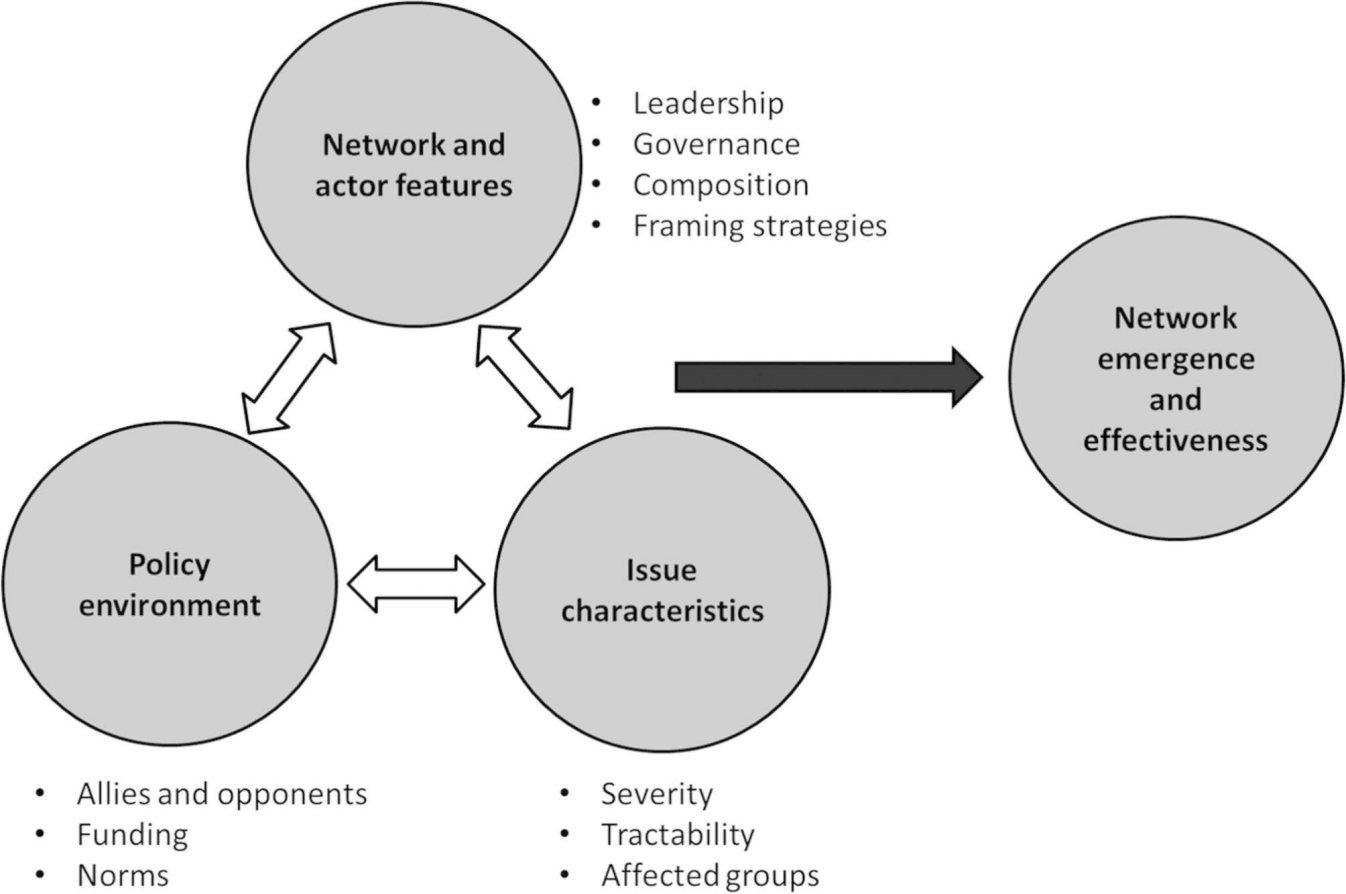
The framework on the emergence and effectiveness of global health networks [20]

The framework on the emergence and effectiveness of global health networks was used to categorize the themes and categories extracted in this study [20]. This framework (Fig. 1) consists of 10 factors in three categories: (1) features of the network and the actors that comprise them, including leadership, governance arrangements, network composition, and framing strategies; (2) conditions in the global policy environment, including potential allies and opponents, funding availability, and global expectations of which issues should be prioritized; and (3) characteristics of the issue, including severity, tractability, and affected groups [20].

The ethical considerations of this study included: receiving ethical approval from the Iran University of Medical Sciences (code: IR.IUMS.REC.1397.438); obtaining informed consent from the interviewees; allowing the participants to withdraw at any point; maintaining the privacy of their personal information; obtaining permission to record audio; and not involving the researchers’ personal opinions in the stages of data collection, analysis, and reporting.

## Results

A total of 21 interviews were conducted with individuals who met the inclusion criteria. Most participants had a Ph.D. (52.4%) and more than 10 years of work experience (71.4%). The average work experience of the participants was 19 years. All participants agreed to be recorded during the interview. Participants were selected from a range of related disciplines such as health policy, health care management, epidemiology, and other specialties and all relevant levels of diplomacy. The interviews were conducted between October 2019 to July 2020. Each interview lasted between 31 min and 1 h and 37 min, with an average of 45 min and 20 s. According to the framework of global health networks, the findings of the interviews were divided into three general categories, including the features of the network and the actors, the policy environment, and the characteristics of the issue.

### Features of the network and the actors

Actors involved in the NCDs prevention diplomacy are defined in a network of collaborations. All these actors interact with and influence each other at different levels. They also have different characteristics. Based on the information obtained from the interviews, four main features were identified for the NCDs network and actors, including leadership, governance, composition, and framing of strategies (Table 1).

**Table 1:** The participants’ views about the features of the network and the actors

Dimension	Themes	Subthemes
Features of the network and the actors	Leadership	• The key role of the UN and WHO in establishing coordination • WHO’s commitment to strengthening accountability for NCD-related actions and to simplifying and streamlining the mechanisms for doing so • WHO’s commitment to working with all governments and non-state actors • WHO monitoring the key factors in NCDs prevention and control • WHO’s mission to provide technical assistance to health ministries and national institutions in designing and evaluating interventions, policymaking, and advocacy
	Governance	• Governance tasks: research, policymaking, advocacy, planning, resource mobilization, implementation • Proposed structure: A secretariat, a high-level council, an advisory group, and an executive committee • The need for comprehensive planning at higher levels of decision making • Strengthening political commitments at the highest levels to address the priority areas of the NCDs program
	Composition	• Involvement of state actors from all sectors (health and non-health sectors such as the Ministry of Foreign Affairs, Agriculture, Sports, Industry, etc.), NGOs and charities, representatives of patients, and international organizations • Strengthening the role of the private sector in NCDs prevention and control • Changing the general approach of the government and the whole society to health in order to create healthy environments • The need for a national response that engages all the sectors involved
	Framing of Strategies	• Strategy 1: Focusing on people, not the disease • Strategy 2: Promoting healthy products, behaviors, and lifestyles • Strategy 3: Mobilizing resources and promoting investment nationwide to strengthen multisectoral collaborations and partnerships and support the implementation of cost-effective interventions in NCDs programs

From the interviewees’ perspective, one of the key features of the NCDs network of actors is the leadership role. This role is mostly planned by the UN and WHO. Objectives and policies related to NCDs prevention and control, risk factors, interaction with the private sector and NGOs, etc. are developed by WHO and are provided to governments through guides and guidelines. One of the interviewees said that:

“*WHO’s mission is to provide technical assistance to the Ministry of Health and national institutions for designing and evaluating interventions, policymaking, and advocacy. Review and suggestion of preventive measures by WHO to combat premature death and promote lifestyle changes are one of the most important initiatives of this organization and one of our tasks is to inform the public about these preventive measures.”* (I 17).

Also, the government is in charge of NCDs governance in the countries. The highest level of government in each country is responsible for providing policy guidance and garnering the political will to support participation in tackling NCDs. To this end, According to the participants’ opinion, it is important to establish a comprehensive, multisectoral mechanism at the highest level. Based on their opinion, this mechanism has five different responsilitise that affect each other and form a cycle:

1. **Research**: Research in the field of NCDs is typically done for two reasons: for evidence-based policy-making and advocacy. One of the interviewees stated that:

“*By presenting research results to different groups of actors and highlighting the importance of the issue, we can gain the support of influential groups. Research should also be used to prioritize different interventions.*” (I 17)

2. **Policy-making**: Policy-making and the development of evidence-based policies are other responsibilities of this mechanism. All the actors should be involved in this process. One of the interviewees said that:

“*When the groups in charge of implementing policies are involved in developing them, they will cooperate more in the implementation phase. The Ministry of Health can perform the task of bringing together different groups according to the issue at hand, including those affected by NCDs.*” (I 17)

3. **Advocacy**: The issue of advocacy can be approached from two perspectives: highlighting the importance of the problem and attracting the support of different groups. At this stage, in addition to the multisectoral mechanism, the Ministry of Health, NGOs, and patients and their representatives can play an important role.
4. **Implementation**: An important issue in the implementation stage is educating the public. Planning and mobilizing resources are also essential to the proper implementation of guidelines.
5. **Monitoring and reporting**: It is necessary to establish a system for continuous monitoring and improvement of legislative and regulatory capacities. Establishing a mechanism for monitoring NCDs funds in the Planning and Budget Organization will ensure the sustainability of resources in this field.

“*The government should enact laws and regulations for the private sector and pharmaceutical companies relating to public health issues.*” (I 12)

“*Follow-up and monitoring are necessary to ensure compliance with the agreements made between Iran and its neighboring countries like Turkey and Azerbaijan or between the Ministry of Health and other sectors such as sports. There is also a need for global, regional, and national, platforms to address these agreements.*” (I 17)

Participants noted that in order to ensure the integration of activities at all NCDs diplomacy levels, it is important that a multi-sectoral mechanism is implemented at all levels and that multiple negotiations take place in other sectors. For example, NCDs Investment case studies can provide important evidence for advocating with governments and policymakers to support the NCDs programs. In this example, the research results can be used both for advocacy at high levels of policymaking and for advocacy of individual donors. From the participants’ perspective, it can consist of a high-level council, an advisory group, and an executive committee, which play complementary roles.

- **Secretariat**: The secretariat is responsible for developing a work plan, holding meetings and compiling minutes, reporting progress, and Supporting the implementation of the functions of working groups.
- **High-level Council**: This council is at the highest level of implementation and is tasked with providing guidance and garnering the political will to support participation.
- **Advisory group**: This working group holds discussions between stakeholders and other actors in the field of NCDs, including intergovernmental organizations (IGOs), civil society, people with NCDs, international agencies, humanitarian organizations, academia, the private sector, and people with other specialties. The advisory group also provides practical, financial, and technical support for participation. Membership is subject to the resolution of any conflict of interest.
- **Executive group**: This committee is responsible for monitoring implementation and ensuring regulatory compliance.

On the other hand, the health sector is single-handedly responsible for bringing together other actors. However, all sectors must be involved in advocacy. Government policies rely on the ability to mobilize a national response that engages all the sectors involved. Therefore, there is a need for a composition of actors and an overall design that are in line with a comprehensive national response, which requires strong health diplomacy.

“*When a program becomes complex, diplomacy becomes necessary for multisectoral collaboration and effective development and execution of the program.*” (I 7).

Altogether, from the interviewees’ perspective, three general strategies that can be adopted for NCDs diplomacy include:

- Strategy 1. Focusing on people, not the disease: The focus of diplomatic negotiations, especially between officials of countries, should be on the importance of increasing people’s health and preventing and controlling NCDs on a large scale. Using health diplomacy as corrective measures in multilateral negotiations focusing on the prevention and control of NCDs can improve the level of community health. Community members, people suffering from non-communicable diseases, and people who have the motivation to prevent and control non-communicable diseases, such as health professionals, should be at the center of these discourses.

“*We should focus on people, not the disease, and for that, we need to promote the human aspect of the disease and the consultation process in the society.*” (I 20)

- Strategy 2: Promoting healthy products, behaviors, and lifestyles: Many of the individual and social factors related to health are things that can be modified and changed only through comprehensive, intersectoral, and long-term strategies that include various approaches to education and health promotion and disease prevention. It is necessary to adopt a comprehensive approach for the development of all aspects of health diplomacy with the approach of changing the attitude of society towards a healthy lifestyle and using new diplomacy tools including media and social networks.

*“National media should inform people about harmful products and should be forbidden from advertising them.” (I 3) “Every year, we hold a campaign on World No Tobacco Day. Well, this campaign lasts for only a month maximum and is not continuous throughout the year. And because of that, it doesn’t have the desired effect.” (I 3)*

- Strategy 3: Mobilizing resources and promoting investment in nationwide collaborations and partnerships and support the implementation of cost-effective interventions in NCDs programs: WHO has proposed guidelines for NCDs prevention, which discuss the need to reduce salt intake, reduce fat intake, increase physical activity, reduce smoking, etc. WHO emphasizes the prevention and control of NCDs because interventions are cheap and effective. These guidelines should be used to educate the public and should also be communicated to other sectors to find the right solutions.

“*Do doctors have the necessary training for NCDs? When it comes to treatment, yes! But is it the same for prevention and control? They’ve learned the most complex surgeries, but may not be familiar with the term ‘NCDs’. They don’t know how to engage people. Therefore, there must be changes in higher education, both in the medical field and other disciplines. The economic sector should also be actively involved, for example, in taxing tobacco and unhealthy foods.*” (I 8)

Therefore, there is a need for a composition of actors and an overall design that are in line with a comprehensive national response, which requires strong health diplomacy.

### Policy environment

Based on the information obtained from the interviews, the policy environment of NCDs prevention and control is divided into three subthemes: allies and opponents, funding, and norms (Table 2).

**Table 2:** Participants’ views about the policy environment

Dimension	Themes	Subthemes
Policy environment	Allies and opponents	• Key allies o UN and its agencies such as WHO o Ministries/departments of health o NCDs Alliance • Key opponents o Industries such as tobacco and alcohol
	Funding	• Different national priorities • High political power of special interest groups • Income and GDP of countries • Involving the public and private sectors as well as NCDs patients • Increasing domestic funding through innovative mechanisms such as taxes on tobacco, alcohol, and sugar-sweetened beverages • Lack of financial resources, unsustainable financing, and resource mobilization challenges • The budget mechanism is not aligned with the National Development Plan • Government’s dependence on the private sector and taxes on harmful products
	Norms	• Sustainable Development Goals (SDGs) • Political Declaration of the High-Level Meeting of the General Assembly on the Prevention and Control of NCDs • National Committee on NCDs

Participants pointed out that there are many groups whose interests align with the goals of the NCDs network of actors, it is more likely to expand and be more efficient than groups that lack potential allies. On the other hand, the relationship between the opponents and the network is not as simple and must be identified in order to determine the impact of these actors on the network. One of the most important global allies in NCDs diplomacy is the UN and one of its most important agencies, i.e. WHO. These organizations have strong advocacy positions, power, and interests. According to one of the participants, the impact of advocacy by these organizations on Iranian senior officials is high.

“*The presence of WHO representatives and their meeting with our senior officials is much more impactful than when this issue is raised by the lower levels of management.*” (I 4).

Given that NCDs go beyond the health sector and require strong multisectoral collaboration, the position, power, and interests of different sectors are different. In Iran, the Ministry of Health and the Ministry of Foreign Affairs have strong advocacy positions and interests. The Ministry of Health has moderate power, while the power of the Ministry of Foreign Affairs is high. In the case of the Ministry of Industry, power is high, while interest and advocacy positions are weak.

“*Our friends in the Ministry of Industry argue that if we heavily tax certain products, factories will shut down, unemployment will increase, and industries will be disrupted.*” (I 3).

Industries such as tobacco are the main opponents of this network.

“*Sometimes the tobacco industry gets more attention than the health sector.*” (I 6).

On the other hand, Various national priorities compete for resources. Sometimes the priorities are not in line with the goals of NCDs health diplomacy. The political power of some companies in the NCDs political space is a major obstacle to NCDs diplomacy. Therefore, there is active opposition to any collective, multisectoral action and is greatly influenced by these powerful special interests.

The income and GDP of a country are also important factors in its NCDs policy. If a policy is adopted that somehow affects the country’s income and GDP, it may impact the efforts aimed at advancing health goals. Lack of financial resources, unsustainable financing, and resource mobilization for NCDs are major challenges since unsustainable resources mean unsustainable programs.

“*For example, if the name or nature of a food product changes, people may not be as interested in it as before, which may lead to the unemployment of several hundred workers and the consequent reduction in GDP, and industries may use this argument in their negotiations, which could be a major barrier.*” (I 2).

The next important issue in financing that participants pointed out is resource mobilization. Taxes on harmful substances, most importantly tobacco and cigarette, along with fines for driving offenses and health offenses, are the main sources of sustainable funding that must be properly increased and allocated to the executive bodies involved in NCDs prevention and control. Unfortunately, the tax on retail cigarette sales is very low in Iran, *which has rend*ered a huge source of funds moot.Taxes on cigarettes and other harmful goods not only help promote healthy behaviors but also increase the financial resources available to the health sector and other sectors involved in combating NCDs. Another challenge is that the budget mechanism is not aligned with the National Development Plan.

“*1% of the country’s GDP is spent on tobacco instead of infrastructure. Each year, about 10 thousand billion tomans are spent on cigarettes and 2 to three times that is spent on the treatment of smokers. It’s worth noting that these expenses are paid for out-of-pocket.*” (I 17) “*We have to make sure that the budget is needs-based and protects people. It has to be sustainable and cost-effective. In many countries the problem isn’t the amount of money available, but how the money is spent. This depends on governance arrangements, power relations, and negotiation power.*” (I 12).

The dependence of the government on the private sector and taxes on harmful products are other governance challenges. So the government faces a dilemma about competing goals because, on the one hand, it wants to improve public health and prevent NCDs. On the other hand, it tries to maintain its revenue stream from taxes on harmful products to spend on health care, education, courts, and many other things.

*“Government revenue may depend on taxes on cigarette sales.”* (I 19).

Another point is that being on the global agenda such as The Sustainable Development Goals (SDGs) influences the development of global health diplomacy in the prevention and control of NCDs. So SDGs are the main norm for promoting the NCDs network at the global level. In addition, the ‘Political Declaration of the High-Level Meeting of the General Assembly on the Prevention and Control of NCDs’ along with the establishment of a national NCDs committee provides the platform for tackling NCDs through multisectoral and multi-stakeholder collaborations and GHD.

*“Sustainable development is a universally accepted narrative.”* (I 20).

### Characteristics of the issue

Based on the information from the interviews, politicians and health diplomats must pay special attention to NCDs due to the many complexities of these diseases, including their high burden, a multitude of risk factors, and the large number of actors involved in the response to NCDs and their risk factors. Based on the information from the interviews, the characteristics of NCDs can be examined from three aspects: severity, affected groups, and tractability (Table 3).

**Table 3:** Participants’ views about the characteristics of NCDs

Dimension	Themes	Subthemes
Characteristics of the issue	Severity	• High burden of NCDs and premature mortality • Extensive risk factors associated with unhealthy behaviors and lifestyles • Long duration of NCDs • The need for a whole-of-government approach to address all risk factors • NCDs as a global problem • Social and economic impact in the form of productivity loss, absenteeism, lost GDP, etc. • NCDs management, which requires the inclusion of NCDs in benefit packages and service delivery with a particular focus on NCDs
	Affected groups	• People with NCDs • People living with NCDs patients • Peare driven to poverty as a result of NCDs • Harmful commodity industries
	Tractability	• Variety of NCDs and NCDs responses • Attempts to attribute NCDs only to individual behaviors • People’s tendency to not react to or take NCDs seriously • Balancing health promotion, prevention, and treatment and emphasizing early detection

According to the participants’ opinion, one of the inherent characteristics of this issue is the burden of NCDs which is increasing globally and shouldn’t be ignored due to its enormous social and health impact. Reducing the global burden of NCDs is an urgent priority. An important aspect of NCDs is that they’re controllable, but require complex management, and are mostly preventable, but the determinants are very complex.

“*Our policies need to be more complex to account for the complexity of NCDs.*” (I 15).

On the other hand, participants pointed out that the severity of the disease and complications cannot be handled by the Ministry of Health alone. NCDs require a holistic approach and shouldn’t just be limited to health and medical issues. The Ministry of Health, the Ministry of Agriculture, the Ministry of Culture, the Ministry of Sports, and the Ministry of Education must be involved in order to create a comprehensive policy for NCDs prevention and control. A whole-of-government approach shoud be adopted, which means that the whole government system gets involved to be able to intervene in the field of NCDs.

“*The response to NCDs is often very different from the kinds of interventions we’ve used to address other types of diseases.*” (I 19).

The main groups affected by NCDs policies include people who have NCDs or live with those who have these diseases. NCDs push many people into poverty, and in turn, poverty is associated with malnutrition and increases the risk of NCDs. On the other hand, harmful commodity industries are also affected by NCDs policies. For example, people who are engaged in tobacco cultivation are affected by bans on tobacco cultivation and advertising, and by advocacy activities aimed at reducing smoking.

“*NCDs are the biggest killers in the world today, and the burden falls disproportionately on the poor.*” (I 18).

Policy-making in the field of NCDs is complex with a wide variety of related activities. It is necessary to act intelligently and seek appropriate support in order to effectively deal with all the aspects of NCDs. Moreover, the activities of all government agencies directly or indirectly affect NCDs policies. Furthermore, there is a strong effort to attribute NCDs to individual behaviors and personal choices, and there is little public opposition to this effort.

“*Risk factors are traditionally considered to be individual, but that’s not the case, and at the end of the day, we are all products of our societies. If the whole society moves in one direction, it’s very difficult to go the opposite way.*” (I 21).

The existence of different views and competing interests and imbalances in power can cause influence politicians. Therefore, how to create a policy network in interaction with politicians is very important.

## Discussion

Considering that the prevention and control of NCDs require multisectoral collaboration at the national, regional, and international levels, it is important to examine the actions and perspectives of actors involved in GHD for NCDs. The present qualitative study investigated this using the framework of the emergence and effectiveness of global health networks. Three general domains were investigated, including the features of the network and the actors, the policy environment, and the characteristics of the issue.

In this study, the features of the network and the actors involved in NCDs prevention were identified and divided into four main categories: leadership, governance, composition, and framing of strategies. Shiffman has mentioned that “network and actor features” are associated with factors internal to the network, inclusive of strategy and structure, and attributes of the actors that constitute the network or have a function in creating it [20]. The present study showed that the leadership role in the network of NCDs actors is mostly played by the UN and especially WHO. Due to the rising trends in mortality from NCDs, WHO has declared NCDs as one of the top health priorities of countries for the last two decades [2] and has requested policymakers to develop effective strategies to stop NCDs [22].

With the recent efforts of the UN, WHO, and the heads of countries to tackle NCDs at the global level, goals and policies related to the prevention and control of NCDs have been designed, and various mechanisms and guidelines/recommendations have been created that represent the official policy of these are organizations [23]. For example, the Global Coordination Mechanism on the Prevention and Control of NCDs will accelerate the implementation of the WHO Global Action Plan NCDs prevention and control and NCDs-related SDGs by promoting high-level commitments on NCDs at the local, national, regional, and global levels. This global mechanism brings together a wide range of stakeholders, identifies and strengthens partnerships, and seeks to find innovative solutions to reduce NCDs through five key activities: advocating and raising awareness; dissemination of knowledge and information; encouraging innovation and identifying barriers; advancing multisectoral actions; and advocating for mobilization of resources [24].

The next feature of the global NCDs prevention and control network is its type of governance. The highly complex nature of NCDs requires complex strategies that can only be implemented with strong governance. Since the highest level of each country is responsible for developing policy guidelines and garnering the political will to support collaboration in the field of NCDs, a comprehensive multisectoral mechanism should be established at the highest level. The most effective public health interventions consist of an evidence-based technical package that is a combination of actions at different levels that together will lead to progress in the prevention and control of NCDs [25].

**Fig. 2:**
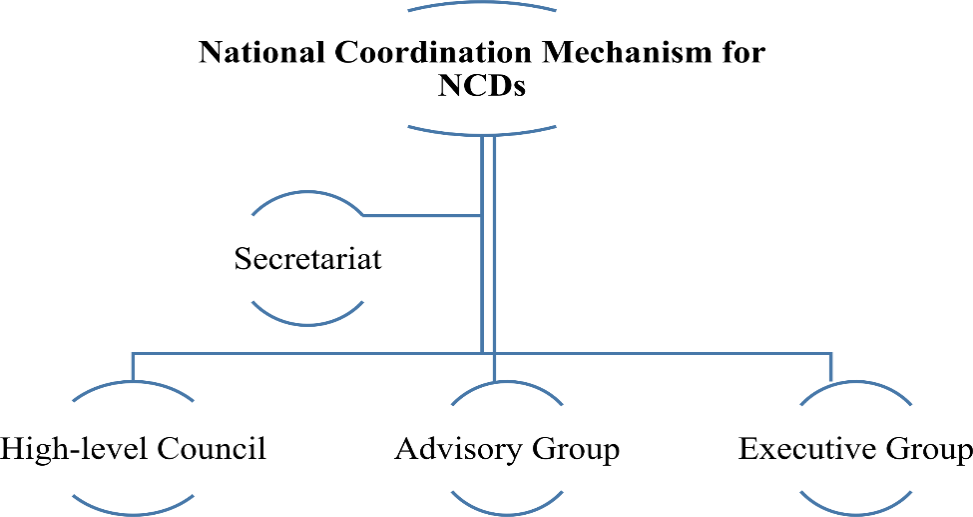
Proposed coordination mechanism for NCDs.

**Fig. 3:**
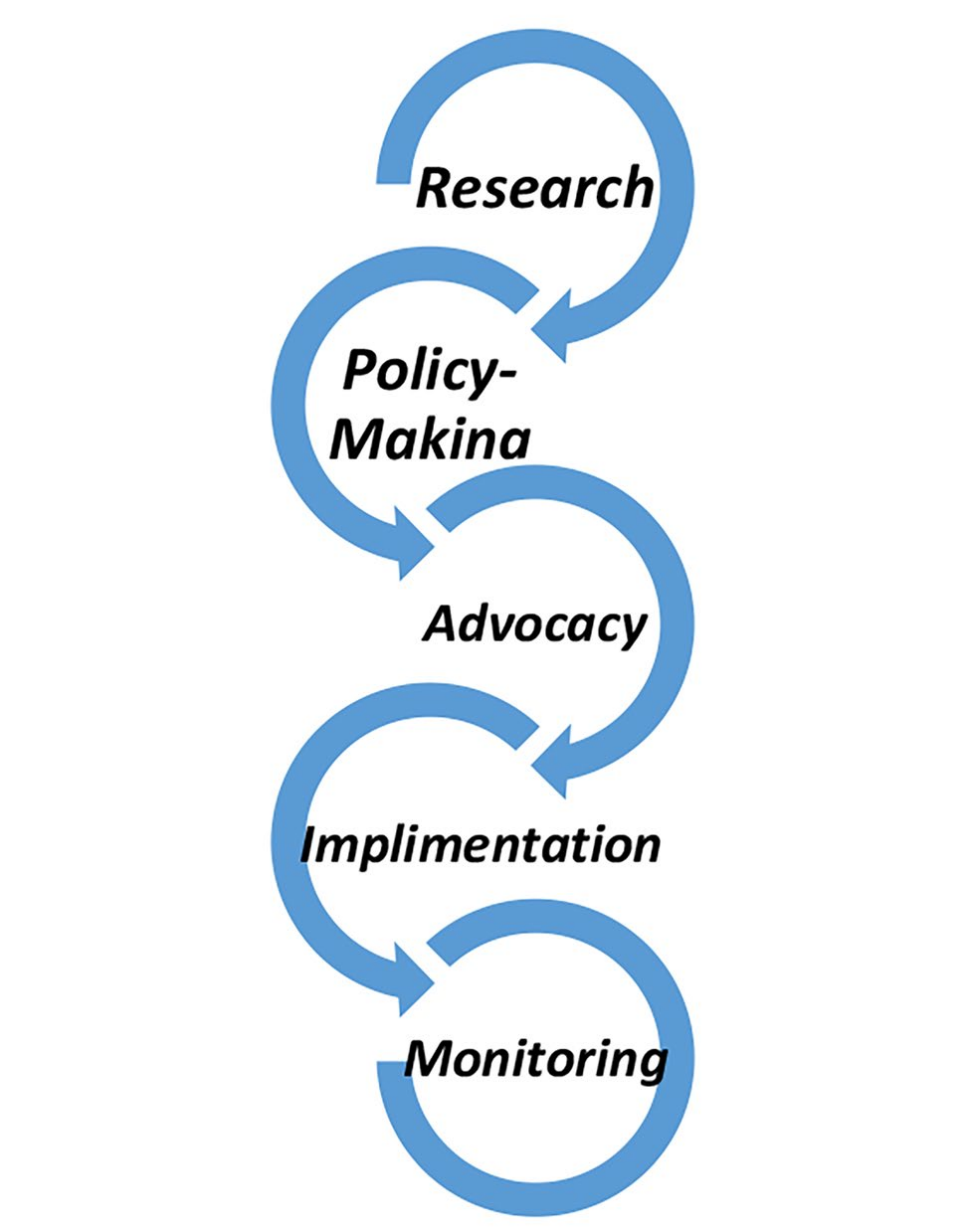
The proposed model for the responsibilities of a multisectoral NCDs mechanism

This study proposes a comprehensive intersectoral mechanism for the governance of NCDs prevention and control at the national level, consisting of a high-level council, an advisory group, and an executive committee (Fig. 2) with five responsibilities: research, policy-making, advocacy, implementation, and monitoring (Fig. 3). Various national mechanisms have been established in different countries. The Health Sciences Authority (HAS) in Singapore, established in 2001, coordinates national health promotion efforts and disease management programs to reduce NCDs by involving multiple sectors [26]. The National Health Commission (NHC) in Thailand is also a cross-sectoral mechanism that is chaired by the Prime Minister and includes three broad sectors—government, academia, and civil society—to emphasize health promotion and support the development of healthy public policies [27].

The next issue in the global NCDs prevention and control network is the composition of actors. Since the health sector alone has the role of calling and bringing together other sectors and requires the advocacy of all actors, the right composition and overall design must be determined in line with a comprehensive national response that also requires strong health diplomacy. Comprehensive multisectoral NCDs action in Iran also requires a collaborative approach that combines the contributions of all relevant stakeholders. Hence, by involving several government agencies, a ‘whole-of-government’ or ‘health in all policies’ approach can be implemented to influence public health policy [8].

A study in India proposes a mechanism for multisectoral action on NCDs that engages various government agencies such as ministries of health, finance, housing, education, agriculture, urban and rural development, transport, commerce, environment, information, and broadcasting. The prime minister is at the core of this mechanism to ensure effective coordination. NGOs link government programs and the community and play an important role in this multisectoral mechanism [28]. The global commitment to tackling NCDs has resulted in the development of strategies such as a multi-stakeholder structure as a holistic platform to enable transparency and accountability to negotiate policy space for NCDs [29].

The tendency to advocate for a whole-of-society approach and promote public-private partnerships without establishing a robust system for managing conflicts of interest is the worst possible combination from the perspective of many NGOs and public health advocates [30]. Given the relative lack of evidence that industry self-regulation or public-private partnerships are effective in reducing NCDs risk factors, the current form of multi-stakeholder collaboration that is guiding global NCDs policies toward voluntary action and public-private partnerships rather than government regulation is worrying [31]. Suzuki et al., in an analysis of the influence of stakeholders on political declarations on NCDs, found that NGOs, academia, and low and middle-income countries (LMICs) countries generally pursue “stricter” governance of NCDs risk factors, while the private sector and high-income countries generally oppose more restrictions on commercial activities and promote a whole-of-society approach that includes cooperation with the private sector [30].

In the present study, three general strategies were identified that the global network and actors in NCDs prevention and control should focus on. The first strategy is that people should be the focus, not the disease. Many NCDs can be controlled by adopting a healthy lifestyle. Community interventions are very useful in reducing these types of diseases because they have a direct impact on everyday life and can be tailored to complex situations [32]. Therefore, community interventions are essential elements in health promotion and NCDs prevention. The effectiveness of interventions has been documented at different ages [33, 34], in different community settings [35], and in different countries [36], for various risk factors such as obesity [37, 38], and public participation in vaccination programs [39].

The second strategy is to continuously inform people about healthy products, behaviors, and lifestyles. In this regard, two distinct and effective functions can be defined for GHD in the area of NCDs prevention and control. Raising awareness, changing discourses, and adopting better educational approaches can change attitudes and increase public demand on the agenda and policies of countries. Therefore, it is possible to influence health policies by promoting health literacy and advocacy by social actors such as NGOs. On the other hand, increased awareness and lifestyle changes will enhance public health in the country. In addition, NGOs and civil society organizations play a prominent role in promoting NCDs prevention and control policies by producing evidence, advocating, and providing technical and financial assistance [16]. Therefore, it is necessary to adopt a comprehensive approach to the development of informal health diplomacy by changing attitudes and promoting healthy lifestyles, and using new diplomacy tools such as social media and social networking.

The third strategy relates to resource mobilization and promotion of investment at the national level to strengthen multisectoral collaborations and support the implementation of cost-effective interventions in NCDs programs. Prioritizing affordable, actionable, fair, and evidence-based interventions will bring the highest return on investment for public health in line with national priorities and will put countries on the path to achieving SDGs [40]. Jackson-Morris and Nugent provide a model of support for national NCDs policy and program in which investment is a key component. This model emphasizes working with national stakeholders to select the most effective and cost-effective policies and programs for a specific national context, identifying sustainable sources of funding, and creating a costed and actionable implementation plan [41], which are consistent with the present findings.

The second dimension in the framework of the emergence and effectiveness of the global NCDs network is the policy environment. The information obtained from the interviews regarding the policy environment and the diplomacy context for NCDs prevention and control was categorized into three main themes: allies and opponents, funding, and norms. The policy environment relates to factors external to the network that shape both its nature and the effects that the network hopes to produce. It is assumed that networks do not operate in a vacuum; rather, they are shaped by outside forces [20].

The findings of this study show that allies and opponents are very influential in the emergence and effectiveness of the global NCDs network. This is consistent with the results of Suzuki et al. regarding the influence of stakeholders on the UN’s political declarations on NCDs [20]. They found that many of the issues mentioned in these declarations are highly contested among stakeholders and tend to be included in the declaration using vague language. Drug prices and regulation of harmful products are among the areas of ​​disagreement among stakeholders [20].

Other studies have also shown that public health advocates emphasize the social and commercial determinants of health and the role of public regulations. Meanwhile, the private sector seeks to promote market-oriented policies backed by neoliberal assumptions [41–44]. According to Suzuki et al., there is so much ambiguity regarding the actors involved in NCDs diplomacy that the Political Declaration of the Third High-level Meeting (HLM) on the Prevention and Control of NCDs (2018) merely uses the term “conflict of interest” without specifying how to manage it [20].

According to the present findings, the financial resources of a country are used for competing priorities, which are sometimes not in line with the country’s NCDs diplomacy goals. In terms of allocation of funds and implementation, there is poor coordination between health-related policies and development policies. NCDs-specific programs do not necessarily expand along the lines established in the national health plan and may be completely independent. Moreover, the development of NCDs-specific programs is in most cases coordinated by the Ministry of Health, and the role and influence of the country’s planning and policymaking units in the development of these policies is not completely clear. An important factor in ensuring the implementation of NCDs policies and programs is the level of political consensus and commitment. This is achieved by involving key stakeholders in the preliminary stages of NCDs strategies [45].

The next issue in shaping the NCDs policy environment is norms. Widely held expectations that global actors address a specific condition are influential in the emergence of the network [20]. According to the present findings, the main norms affecting the global NCDs network are SDGs. SDG Target 3.4 calls for reducing premature death from NCDs, which has been neglected in the Millennium Development Goals (MDGs) [46]. However, the achievement of both MDGs and SDGs depends on NCDs prevention and control, which highlights the importance of coordinated action on NCDs at local, national, and global levels.

In addition, governments should interact with the private sector, academia, civil society, and the general public to develop community-based models of NCDs prevention and control that take into account experiences and challenges. Efforts should be made to create healthier environments through effective regulations based on national health priorities with people’s participation and with clear objectives. Interactions should be transparent and include accountability, evaluation, and specific time frames. The government should engage the private sector and look for ways to strengthen commitments and help achieve the goals of the health system [47, 48]. Participation of civil society organizations, the private sector, the media, and donor organizations is equally important because the policies and programs that are carried out to promote health must be widely accepted in order to be successful [49].

The third dimension in the framework on the emergence and effectiveness of the global NCDs network relates to issue characteristics, which are the features of the problem the network tries to address. This dimension is based on the idea that issues vary in several characteristics that make them more or less difficult to tackle [20]. Among the challenges in tackling NCDs are the complications of these diseases, the multitude of risk factors from global (international fast food companies) to local (unpaved roads), and the fact that not all NCDs are preventable. Due to these complexities, it is difficult to set targets and finance them. The global response to NCDs should focus on generating multisectoral evidence on the transnational factors contributing to the increase of NCDs and the potential impact of the policies proposed to control them [5, 47, 48].

In addition, poverty is closely linked with NCDs. Bloom et al.’s study showed that the economic burden of NCDs is estimated to be US$ 81.96 billion for Costa Rica, US$ 18.45 billion for Jamaica, and US$ 477.33 billion for Peru during the period 2015–2030 [50]. The rapid rise in NCDs hinders the success of poverty reduction initiatives in low-income countries, mainly due to the increase in household healthcare expenditure. Vulnerable and socially disadvantaged people get sick more often and die earlier than people of higher social status, especially because they are more exposed to harmful products such as tobacco or unhealthy food and have limited access to health services. In resource-poor settings, healthcare expenditure for cardiovascular disease, cancer, diabetes, or chronic lung disease can quickly deplete household resources, pushing a family into poverty. The exorbitant costs of NCDs, including often lengthy and expensive treatment, together with the loss of income, push millions of people into poverty every year and stifle development.

Effective management of NCDs is complex and costly and requires the contribution of a wide range of actors. Given the increasing pressures that developing countries face with unprepared systems and economies, diverse partnerships may be considered a more important component in the prevention and effective management of NCDs [51]. Since global health networks will be effective when they create compelling framings of the issue and build political coalitions that extend beyond the health sector [52], it can be inferred that to increase the effectiveness of the NCDs prevention and control network, a more comprehensive approach focused on the three dimensions proposed in the Shiffman’s model can be helpful.

Increasing interaction in strengthening multilateral cooperation and networking at the national and transnational level creates sufficient ability and power to improve and promote negotiations for the benefit of health. The non-communicable diseases diplomacy network can help coordinate and raise the priorities of non-communicable diseases. Conducting case studies in the future, using the network approach to promote health diplomacy regarding tobacco control policies, nutrition and physical activity in Iran can help to develop relationships and advance health goals through creating lessons learned for increasing the effectiveness of these networks.

On the other hand, building strong educational and research connections between collaborative networks of physicians and healthcare professionals could be an ideal means of delivering health diplomacy. Therefore, it is suggested to create a network of domestic academics and researchers for national and transnational studies with the approach of capacity building, creating evidence for policy making and training health diplomats. The purpose of creating such a network is to expand the exchange of evidence-based knowledge and information with other countries regarding non-communicable diseases by creating a cooperation network between specialized organizations and researchers to model international health achievements and model national achievements at international levels.

## Conclusion

The findings of the present study suggest that a successful and sustainable NCDs program requires the participation of multiple actors from the government, the private sector, and civil society at international, national, and local levels. The global NCDs prevention and control network should not only highlight the need to improve the factors internal to the network like the relationships among actors but also improve influential external factors such as the policy environment and the complex characteristics of NCDs. Therefore, to reduce the burden of NCDs, in addition to advocacy and building coalitions with civil society, it is essential for health policymakers to use negotiation and diplomacy in their interactions with trade policymakers regarding the health effects of international trade agreements and to manage the conflict of interests of industries.

## Electronic supplementary material

Below is the link to the electronic supplementary material.

Supplementary Material 1

## Data Availability

Not applicable.

## List of abbreviations

NCDs: noncommunicable diseases
GHD: global health diplomacy
UNITAF: United Nations Interagency Task Force on NCDs
WHO: World Health Organization
NASBOD: National and Sub-national Burden of Disease
STEPS: WHO STEPwise approach to Surveillance of NCD risk factors
LMIC: low and middle-income countries
NGOs: non-governmental organizations
HLM: High-level Meeting
INCDC: Iranian National Non-Communicable Diseases Committee
SDGs: Sustainable development goals
