# Supplementary material for: Enhancing global health diplomacy for non-communicable diseases: application of the global health network framework
Source: Global Health. 2023 Jun 21;19:41. doi: 10.1186/s12992-023-00944-7 (PMC10283340; doi:10.1186/s12992-023-00944-7)
Supplement: Supplementary file 1 — Supplementary Material 1 [file 12992_2023_944_MOESM1_ESM.docx]

Point-by-point response:

| Reviewer | **Reviewer comment (s)** | **Response** | **Locates of manuscript** |
| --- | --- | --- | --- |
| Reviewer #1 | - Please replace ‘climate’ with a more specific term. Is this an institutional context? Social, political, and economic contexts? | Thanks to the respected reviewer,  It is done. | Abstract |
|  | - P. 5, line 1 – please edit ‘… the WHO known Iran as a fast-track country …’ to ‘the WHO recognized/designated/named (one of these) Iran as a fast-track …’ | It is done. | Introduction |
|  | - P. 5, para 1 – please explain how and why GHD is crucial to advance NCD prevention in Iran. How will the collaborations that you’ve indicated support this effort? | It is done. | Introduction |
|  | - P. 17, para 1 – Rather than ‘behavior of actors’ is there another way to characterize this emphasis? Perhaps the actions and perspectives of actors? Because your findings identify institutions, processes, etc I think it’s limiting to focus on behavior at the start of this paragraph. | It is done. | Discussion |
|  | - P. 19, line 2 – I suggest removing ‘excellent’ from ‘excellent strategies’. Unless you go on to evaluate the effectiveness of these strategies, I don’t think it can be said that they are good or bad. | It is done. | Discussion |
|  | - P. 22, line 45 – I am unclear how the statement ‘who see peace as a rational public health outcome …’ relates to the study. I suggest removing this point or providing justification and explanation of this point. | It is done. | Discussion |
